# 

**Published:** 1852-06

**Authors:** 


### CONTENTS

### OF NO. VI.

---

### ORIGINAL COMMUNICATIONS.

### ESSAYS AND CASES.

Art. I.—On the Medical Qualities of the Asclepias Syriaca. By George W. Allen, M. D., of Miss. - - - - - 461
Art. II.—On the Treatment of Enlarged Spleen. By W. Y. Gadbury, M. D., of Benton, Miss. - - - - - 466
Art. III.—A Case of Secondary Syphilis. By Robert Durrett, M. D. - - - - - - - - - - - 469
Art. IV.—On the Use of Oxide of Silver. By J. V. Withers. 471

### REVIEWS.

Art. V.—Essays on Life, Sleep, Pain, etc. By Samuel H. Dickson, M. D. - - - - - - - - - 473
Art. VI.—Report of the Committee on Medical Education, appointed by the American Medical Association. By W. Hooker, M. D- - - - - - - - - - - - 495
Art. VII.—Anesthetics. - - - - - - - - - 507

### SELECTIONS FROM FOREIGN AND HOME JOURNALS.

Observations upon the Inutility of the Abdominal Bandage after Parturition, - - - - - - - - - - - 521
Five Calculi Removed by Lithotomy, each containing a Field Bean as a Nucleus. - - - - - - - - 525

Dysentery, complicated with Gastritis. - - - - - - 526
Dropsy of the Amnion. - - - - - - - - - 529
Medical Witnesses and Lawyers. - - - - - - 530
Dysentery in California. - - - - - - - - 531
Mortality in the City of New Orleans. - - - - - 532
Case of Punctured Wound of the Abdomen, involving the Intestines. - - - - - - - - - 533
New Views on Respiration. - - - - - - - 535
External Use of Chloroform. - - - - - - - 536
Professor Christison's Lecture on the present state of Medical Evidence. - - - - - - - - - 540

ORIGINAL INTELLIGENCE.

American Medical Association. - - - - - - - 545

